# Case-Fatality Ratio of Enteric Fever: Estimates From Multitiered Surveillance in India

**DOI:** 10.1093/infdis/jiab388

**Published:** 2021-11-23

**Authors:** Prasanna Samuel, Swathi Krishna Njarekkattuvalappil, Dilesh Kumar, Reshma Raju, Jason R Andrews, Gagandeep Kang, Jacob John

**Affiliations:** 1 Christian Medical College, Vellore,India; 2 Stanford University School of Medicine, Stanford, California,USA

**Keywords:** case-fatality ratio, disease burden, enteric fever, tertiary care surveillance

## Abstract

**Background:**

The case-fatality ratio (CFR) for enteric fever is essential for estimating disease burden and calibrating measures that balance the likely health gains from interventions against social and economic costs.

**Methods:**

We aimed to estimate the CFR for enteric fever using multiple data sources within the National Surveillance System for Enteric Fever in India. This surveillance (2017–2020) was established as a multitiered surveillance system including community cohorts (tier 1), facility-based (tier 2), and tertiary care surveillance (tier 3) for estimating the burden of enteric fever in India. The CFR was calculated after accounting for healthcare-seeking behavior for enteric fever and deaths occurring outside the hospital.

**Results:**

A total of 1236 hospitalized patients with blood culture–confirmed enteric fever were enrolled, of which 9 fatal cases were identified, for an estimated hospitalized CFR of 0.73% (95% confidence interval [CI], .33%–1.38%). After adjusting for severity, healthcare-seeking behavior, and deaths occurring out-of-hospital, the CFR was estimated to be 0.16% (95% CI, .07%–.29%) for all enteric fevers.

**Conclusions:**

Our estimates of the CFR are relatively lower than previously estimated, accounting for care-seeking behavior and deaths outside the hospital.

Case-fatality ratio (CFR) provides a critical summary statistic that characterizes disease severity by estimating the proportion of deaths among cases [1]. Estimates of CFRs for enteric fever are limited but required to raise awareness of enteric fever burden, guide priorities for the use of scarce resources, compare health system metrics, and inform the introduction of prevention and control strategies. While a number of studies have estimated the CFR for enteric fever, they have varied substantially across the geographical locations and are constrained by the inherent challenges in its measurement [2–3]. The Global Burden of Disease study, through the notifiable disease data and facility-based reporting, estimates the mean all-age CFR of enteric fever to be 0.95% [4]. A systematic review and meta-analysis on CFRs of enteric fever reported a summary estimate of 4.45% (2.85%–6.88%; n = 21) for hospitalized patients and 2.49% (95% confidence interval [CI], 1.65%–3.75%; n = 44) overall [5]. However, these estimates are derived mostly from older studies, many of which are small and only included patients who sought healthcare and were admitted to hospitals. Because these are facility-based studies, they cannot capture cases that do not seek healthcare due to milder symptoms or for some other reason. Therefore, these studies are limited by the underascertainment of cases (denominator) and deaths caused by enteric fever (numerator), but in general were likely biased toward estimating a CFR for more severe cases that were identified through hospital-based surveillance.

It is well recognized that CFRs calculated from incomplete reporting of both numerator and denominator will lead to biased estimates. The number of diagnosed clinical cases of enteric fever might only represent a fraction of actual infections. Thereby the resulting clinical CFRs are likely to be higher than the actual infection fatality ratio. Similarly, hospitalized cases might represent only a fraction of diagnosed cases with hospitalized CFRs higher than the clinical CFR, which highlights the impact of surveillance biases on CFRs. Unbiased estimation of CFRs requires that recorded number of cases and deaths be representative of all cases and deaths; reliable data are challenging to collect. In many settings, enteric fever may go untreated either because of milder symptoms or the lack of access to healthcare facilities.

Furthermore, fatalities might have occurred without being captured in the surveillance because they occurred outside the facilities. Data on care-seeking behavior and the proportion of deaths among nonhospitalized patients are necessary to account for enteric fever cases that did not come under medical attention. This study aimed to estimate CFRs for enteric fever by integrating multiple data sources within a 3-tiered multicentric surveillance of enteric fever conducted for 2 years in India. The primary goal of this study is to estimate the probability of death for a case of enteric fever.

## METHODS

### Overview

We estimated the CFR for enteric fever assuming that a general population will move at least through 3 levels: (1) enteric fever cases seeking medical attention; (2) deaths among medically attended; and (3) deaths occurring outside the hospital. Under these levels, we broke down the CFR into respective components for which data are available: the probability of hospitalization given enteric fever, and the probability of death given hospitalization. Also, this relation might not hold strictly given that deaths could occur outside the hospital. So we assumed, as part of the third level, the total number of deaths as a sum of those hospitalized and occurring outside of hospital. Furthermore, we used data from 3 tiers of enteric fever surveillance to estimate these probabilities at these successive levels.

### Data Sources

The National Surveillance System for Enteric Fever in India was established as a multitiered, multicentric surveillance system to measure the burden of enteric fever in India. The protocol has been published previously [6] but is briefly described here.

**Tier 1 Data**

The tier 1 active surveillance was carried out in 3 urban or semiurban sites (Delhi, Kolkata, Vellore) and 1 rural site (Pune), recruiting 24 000 children aged 0.5–15 years into closed cohorts for fever surveillance for 24 months. All children with fevers of >3 days received a blood culture, processed in an automated system, and all confirmed cases of enteric fever were followed up daily till the resolution of the episode. This community-based surveillance provided the proportion of enteric fever cases that are hospitalized.

**Tier 2 Data**

The tier 2 facility-based surveillance for febrile illnesses in smaller hospitals was conducted at 6 (5 rural and 1 urban low-income) sites coupled with healthcare utilization surveys of the catchment area. All admissions based on fever received blood cultures and were followed until discharge from the facility. Moreover, all confirmed enteric fever cases were followed up telephonically to determine their health status concerning the current illness episode. The healthcare utilization survey nested in this tier provided an estimate for the proportion of individuals with enteric fever who sought care at healthcare facilities. It also provided the ratio of hospitalized vs nonhospitalized deaths among all febrile illnesses [7].

**Tier 3 Data**

Tier 3 was a laboratory surveillance network from 8 tertiary care centers providing data on blood culture–confirmed enteric fever cases identified from the microbiology laboratories. All culture-confirmed enteric fever inpatients were followed up till discharge and recontacted on the 28th day postdischarge telephonically to inquire about health status concerning the current illness. The surveillance in different tiers was initiated at different times, but each site conducted surveillance for at least 24 months between October 2017 and April 2020. More than 95% of cultures were done by an automated method, and the rest by conventional methods [6, 7]. Data on the clinical course, the outcome of the episode of illness, antimicrobial resistance pattern, and cost of illness were capture for enteric fever cases of all age groups (>6 months of age).

### Ethical Considerations

Informed consent was obtained from all participants. The study protocol was approved by the Institutional Review Board of Christian Medical College, Vellore. India.

### Data Analysis

The CFR was defined as the product of the hospitalized CFR dying (D|H)) and the probability of enteric fever case being hospitalized (H|EF): Case−fatality ratio = P(D|H).P(H|EF) (Table 1). However, deaths could also occur outside the hospital, and so we calculated the total CFR as the sum of hospitalized CFR, P(D|H).P(H|EF), and the probability of deaths among nonhospitalized P(D|NH).P(NH|EF) enteric fever cases (Table 1).

**Table 1.** Abbreviations in the Case-Fatality Ratio Calculation

D	Deaths
H	Hospitalized case(s)
NH	Nonhospitalized case(s)
EF	Enteric fever
P (D|H)	Probability of deaths among hospitalized enteric fever patients
P (D|NH)	Probability of deaths among nonhospitalized enteric fever patients
P (H|EF)	Probability of hospitalization among enteric fever patients
P (NH|EF)	Probability of nonhospitalization among enteric fever patients
r	Ratio of febrile deaths in the hospital compared to febrile deaths outside the hospital

To calculate the probability of deaths among nonhospitalized cases, we considered observed deaths within the hospital as some multiple of deaths outside the hospital, that is,


P(D|H).P(H|EF) = r * P(D|NH).P(NH |EF)

where “r” is the ratio of febrile deaths in the hospital compared to those febrile deaths outside the hospital. Therefore, solving the above for probability of death among nonhospitalized, we have:


P(D|NH) = P(D|H). P(H|EF) / (r*P(NH|EF))

We estimated “r” using available data from the healthcare utilization survey to identify deaths due to fever and the proportion that occurred while hospitalized and outside of the hospital admissions (Table 2).

**Table 2.** Hospitalizations and Deaths From Different Tiers of Surveillance Used for Estimating the Case-Fatality Ratio for Enteric Fever

Enteric Fever Hospitalizations (Tier 1: Active Community Surveillance)			
Study site	Total Enteric Fever, No.	Total Hospitalized, No.	Rate, no./No. (95% CI)
Delhi	80	11	13.8 (7.1–23.3)
Kolkata	94	13	13.8 (7.5–22.5)
Pune	11	8	72.7 (39–94)
Vellore	147	21	14.3 (9–21)
Pooled	332	53	15.9 (12.2–20.4)
Febrile Deaths at the Hospital (Tier 2: Healthcare Utilization Survey Data)			
Study site	Nonhospital Deaths, No.	Hospital Deaths, No.	Ratio, no./No.
Chandigarh	1	11	11
Himachal Pradesh	2	6	3
Bihar	9	24	2.67
Assam	10	13	1.3
Maharashtra	2	8	4
Andhra Pradesh	1	10	10
Average	25	72	2.88
Enteric Fever Deaths (Tier 3: Tertiary Care Surveillance)			
Study site	Total Hospitalized Enteric Fever, No.	Total Deaths, No.	Rate, no./No. (95% CI)
All India Institute of Medical Sciences, Delhi	46	0	…
Post Graduate Institute of Medical Education & Research, Chandigarh	76	4	5.3 (1.5–12.9)
Christian Medical College, Ludhiana	142	1	0.7 (.01–3.9)
Topiwala National Medical College–B. Y. L. Nair Hospital & Kasturba Hospital, Mumbai	66	0	…
St Johns Medical College, Bengaluru	187	2	1.1 (.1–3.8)
Christian Medical College, Vellore	118	0	…
Chacha Nehru Bal Chikitsalaya, Delhi	177	1	0.6 (.01–3.1)
Kanchi Kamakoti Childs Trust Hospital, Chennai	148	0	…
Tier 2 secondary hospitals	276	1	0.3 (.01–2)
Average	1236	9	0.73 (.33–1.38)

Abbreviation: CI, confidence interval.

A Monte Carlo simulation was used to estimate the uncertainty (95% CI) in the CFR. Also, we performed 1-way sensitivity analysis by repeating all calculations and varying “r”—the ratio of deaths in-hospital to outside the hospitals.

## RESULTS

### Hospitalized CFR

The tier 2 surveillance provided data on 276 blood culture–confirmed enteric fever cases. A total of 15 (5.43%) patients developed complications, with hepatitis (n = 4) being the commonest complication. Of the 276 enteric fever patients, 250 (90.6%) recovered without complications, 4 left against medical advice, 21 (7.6%) were referred to other hospitals, and 1 died of complications.

**Figure 1.**
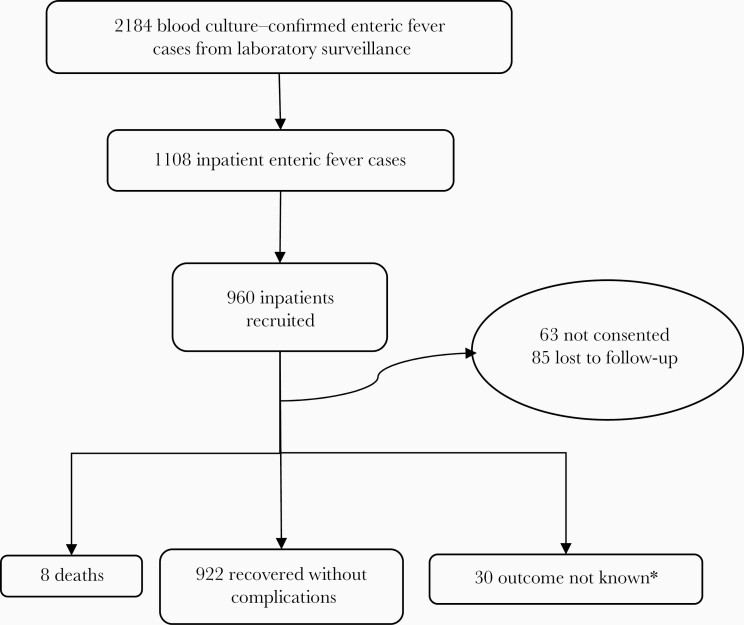
Outcomes among hospitalized enteric fever cases in tertiary care surveillance. *Outcome not known due to leaving against medical advice or referral to other hospitals.

The tier 3 tertiary care surveillance provided data on 960 hospitalized blood culture–confirmed enteric fever cases. Thirty-nine of the 960 (4.06%) recruited patients developed complications during the course of hospitalization, including hepatitis (n = 13), hemodynamic shock (n = 7), gastrointestinal bleed (n = 4), renal impairment (n = 3), intestinal perforation (n = 3), encephalopathy (n = 3), and myocarditis (n = 1). Whereas 922 patients recovered without complications, 26 left against medical advice, 4 were referred to other hospitals, and 8 patients died (7 deaths among patients with typhoid fever, and 1 among those with paratyphoid fever) (Figure 1).

The CFR among hospitalized enteric cases was calculated combining both tier 2 and tier 3 surveillance and was estimated to be 0.73% (n = 9/1236; 95% CI, .33%–1.38%). The demographic and clinical characteristics of the tier 3 cases are presented in Table 3.

**Table 3.** **Characteristics of Hospitalized Culture-Confirmed Enteric Fever Cases and Deaths From Tertiary Care Surveillance** ^a^

Characteristic	Culture-Confirmed Cases Excluding Deaths (n = 952)	Deaths (n = 8)
Age, y, mean (SD)	13.8 (12.6)	26.9 (24.7)
Age category		
Pediatric (<15 y)	600 (63%)	4 (50%)
Adults (≥15 y)	352 (37%)	4 (50%)
Sex		
Male	587 (62%)	6 (75%)
Female	365 (38.3%)	2 (25%)
Duration of fever, d, median (range)	13 (1–189)	15 (4–27)
Duration of hospital stay, d, median (range)	7 (1–40)	6.5 (2–40)
Presenting symptoms		
Fever	946 (99.4%)	7 (87.5%)
Vomiting	463 (48.6%)	5 (62.5%)
Abdominal pain	378 (39.71%)	4 (50%)
Diarrhea	288 (30.3%)	5 (62.5%)
Cough	225 (23.6%)	2 (25%)
Headache	212 (22.3%)	3 (37.5%)
Highest temperature recorded during the illness episode, °C, mean (SD)	39.45 (0.77);	39.65 (0.98)

Data are presented as No. (%) unless otherwise indicated.

Abbreviation: SD, standard deviation.

^a^One death from the tier 2 secondary care surveillance is not presented here

### Probability of Hospitalization Among Enteric Fever Cases

The tier 1 surveillance, based on a community cohort, provided data on 24 000 children. It was estimated from this surveillance that the probability of hospitalization in those with culture-confirmed enteric fever, with a fever for at least 3 days, was 0.159.

### Ratio of Deaths Occurring Inside the Hospital Compared With Outside the Hospital

With data from the healthcare utilization survey, we estimated the ratio of in-hospital deaths to deaths outside the hospital as 2.88 (72/25). We then calculated the CFR for nonhospitalized enteric fever cases as 0.048%.

### Clinical CFR

Combining the hospitalized and nonhospitalized CFRs, weighted by the proportion of febrile cases hospitalized, we estimated the overall CFR to be 0.16% (95% CI, .07%–.29%). In sensitivity analysis varying the ratio of deaths occurring in-hospital vs outside the hospital from 6 (twice the observed) to 1.5 (half the observed), the overall estimated CFR varied between 0.13% and 0.19%.

## Discussion

Using data from our tiered surveillance for enteric fever, we have estimated an overall CFR of 0.16%, a weighted estimate comprised of a CFR of 0.73% for patients requiring hospital admission and 0.048% for patients managed outside the hospital. These estimates were derived from hospitalized cases and data from healthcare utilization surveys used as ancillary sources to adjust for healthcare-seeking behavior and out-of-hospital deaths. Within the assumption made regarding the proportion of out-of-hospital deaths, these estimates are uncertain up to a factor of 1.2, as reflected in the sensitivity analysis associated with our estimates.

Our estimate of CFR is substantially lower than those provided by Pieters et al [5], which range from 1.68% to 3.49% overall, and from 2.88% to 6.88% among hospitalized patients. However, authors discussed the issue of heterogeneity in CFRs across studies that could not be explained by any single factor owing to larger differences in disease management, culture, access to care, and so forth. More recently, Yu et al [8] estimated that between 0.05% and 0.55% of laboratory-confirmed cases in Dhaka, Bangladesh, were fatal. Our estimates fall within the lower part of this range. Crump et al [9], on the other hand, estimated a 1% CFR from hospital-based studies, which was similar to our estimates among hospitalized cases.

Obtaining an accurate estimate of the enteric fever burden is challenging, given that only a proportion of these cases present to medical care. In the absence of ancillary or enhanced studies, most cases might go unaccounted for and thus might lead to biased estimates of disease burden. One of our study’s strengths is that the estimate of CFR was derived by combining different data sources available within the tiered enteric fever surveillance system. This broader approach enabled us to overcome limitations associated with using either a community- or hospital-based approach [10]. While a community-based approach captures all cases, including the milder ones that would generally not present to the hospital, they alter the natural course of disease due to early detection and treatment and falsely reduce CFRs. Nonetheless, we were able to obtain an estimated rate of hospitalization in the pediatric cohort. This may have been a lower rate of hospitalization because of early treatment, but would be expected to be higher than would be seen in the adult population. Hence we chose to retain the 0.159 estimate and apply it to all ages to account for the potential for both under- and overestimation. On the other hand, hospital-based approaches offer a simpler alternative, but tend to be biased toward more severe cases and toward those who could seek medical attention. We were able to account for out-of-hospital death by deriving a ratio of hospitalized to nonhospitalized fever deaths in the community using data from healthcare utilization survey. By combining data from both community and hospital surveillance, and using healthcare utilization surveys as an ancillary data source, our study sought to improve estimates of CFR. The limitations of the study include the few deaths on which CFR has been calculated, which precluded stratification of CFR by age or typhoidal *Salmonella* serotype.

## Conclusions

Our study shows that enteric fever continues to be a public health problem with substantial case-fatality in low-middle-income countries. Our findings highlight the value of a multitiered surveillance approach and inform model parameters for typhoid burden and cost-effectiveness of intervention measures.

## Notes

***Author contributions.*** J. J. and J. R. A. conceived of and designed the study. J. J., P. S., J. R. A., and G. K. developed the study protocol and analysis plan. J. J. and G. K. coordinated the project. P. S., S. K. N., D. K., and R. R. coordinated data collection at the sites. P. S. and S. K. N. analyzed the data. P. S., S. K. N., and D. K. drafted the manuscript. All authors have reviewed and approved the manuscript. J. J. and G. K. had complete access to data and guarantee the manuscript.

***Acknowledgments.*** We acknowledge with gratitude the participants and their families for their partnership over the 2 years of surveillance. We acknowledge all Surveillance for Enteric Fever in India collaborators for their support at various stages of the surveillance.

***Supplement sponsorship.*** This supplement is sponsored by the Christian Medical College Vellore Association.

***Disclaimer.*** The funder had no role in the design analysis or interpretation of the findings.

***Financial support.*** This study was part of the National Surveillance System for Enteric Fever in India, supported by a grant from the Bill & Melinda Gates Foundation (Investment ID–INV-009497-OPP1159351).

***Potential conflicts of interest.*** All authors: No reported conflicts of interest.
